# A triple protein-based ELISA for differential detection of ASFV antibodies

**DOI:** 10.3389/fvets.2024.1489483

**Published:** 2024-12-11

**Authors:** Shuai Zhang, Yuzhu Zuo, Wenyuan Gu, Yunhuan Zhao, Ying Liu, Jinghui Fan

**Affiliations:** ^1^College of Veterinary Medicine, Hebei Agricultural University, Baoding, China; ^2^Hebei Animal Disease Control Center, Shijiazhuang, China; ^3^Hebei Veterinary Biotechnology Innovation Center, Baoding, China

**Keywords:** African swine fever virus, indirect ELISA, differential diagnosis, wild-type strain, gene-deleted strain

## Abstract

African swine fever (ASF) caused by the ASF virus (ASFV) is a severe and highly contagious viral disease that poses a significant threat to the global pig industry. As no vaccines or effective drugs are available to aid prevention and control, early detection is crucial. The emergence of the low-virulence ASFV strain not expressing CD2v/MGFs (ASFVΔCD2v/ΔMGFs) has been identified domestically and internationally and has even become an epidemic in China, resulting in a complex epidemic. The commercialized ASFV ELISA kits available can detect the presence of ASFV infection in pigs, but they are unable to distinguish wild-type ASFV from gene-deleted strains. The current published ELISA assays can distinguish between the wild-type and CD2v gene-deleted ASFV but cannot differentiate wild-type and MGF505 gene-deleted ASFV or CD2v and MGF505 double-gene deleted ASFV infection, posing new challenges for an effective prevention and control of ASFV. In this study, the ASFV-p30, ASFV-CD2v, and ASFV-MGF505 proteins were expressed using a prokaryotic expression system, and a triple protein-based ELISA antibody detection method based on these proteins was successfully established to effectively differentiate between wild-type ASFV and ASFVΔCD2v and/or ASFVΔMGF505 virus infection. This triple protein-based ELISA showed good analytical specificity without cross-reactivity with antibodies against PRRSV, CSFV, PRV, and PCV2. Moreover, it demonstrates remarkable analytical sensitivity by allowing the identification of clinical samples even at dilutions as high as 1:800. The coefficient of variation the intra-assay and inter-assay were below 5%, indicating strong repeatability and reproducibility. To evaluate the performance of the triple protein-based ELISA, a total of 59 clinical serum samples were detected using the triple protein-based ELISA. The results showed that 22 samples were positive for ASFV, of which 19 were ASFV wild-type, one was ASFVΔCD2v, and two were ASFVΔMGF505. Compared with the commercialized triplex qPCR kit, the triple protein-based ELISA exhibited high diagnostic sensitivity and diagnostic specificity. The test accuracy with the commercialized triplex qPCR kit was 98.31% (58/59), and the test accuracy with the commercialized ELISA kit was 96.61% (57/59). These results indicated that the developed triple protein-based ELISA performs well in detection and differentiation. Therefore, it will be useful for the ASFV serological differential diagnosis and epidemiology study.

## 1 Introduction

African swine fever (ASF) is a highly contagious infectious disease caused by the ASF virus (ASFV) (1). Clinical manifestations range from acute to chronic or asymptomatic (2, 3). Acute ASF caused by highly virulent ASFV (usually named wild-type ASFV), has a fatality rate of 100% (3, 4). Chronic or asymptomatic ASF caused by lower virulent mutant are highly transmissible, can shed ASFV for a long time and infect susceptible pigs via direct or indirect contact (5–8), but not easy to be find clinically. ASFV was first discovered in Kenya in 1921 and has spread to many countries (9–15). Since ASF was introduced in China in August 2018, it quickly spread across pig farms throughout China and led to a sharp decline in pig populations (16). From the end of 2020, naturally attenuated ASFV strains emerged in some domestic pig farms in China and some other ASFV affected countries (17–23). The co-circulating of the wild type ASFV and naturally lower-virulence ASFV complicates the situation of epidemic disease. According to the WOAH reported from January 2020 to January 2022, 35 countries have reported African swine fever outbreaks, resulting in significant losses in domestic pig (1,043,334 animals lost) and wild boar populations (29,970 animals lost) (24). Currently, ASF has spread extensively globally, brings significant economic losses to the pig industry.

The etiological agent, ASFV, is a large double-stranded DNA virus with an envelope and icosahedral structure. The full length of the ASFV DNA genome varies between 170 kb and 193 kb. It encodes about 200 proteins, most of which are related to virus replication, immune escape, virus transmission, and so on (15). The p30 protein encoded by the CP204L gene is a relatively conserved immunogenic capsid protein of ASFV, which can stimulate the body to produce antibodies with certain neutralizing abilities in the early stage of virus infection and last for a long time. It is an ideal antibody diagnostic antigen for ASFV (25, 26). CD2v protein is expressed in the late stage of ASFV infection and encoded by the EP402R gene. Wild-type ASFV infection induces the production of specific antibodies that recognize the CD2v protein. CD2v protein is an important structural surface antigen of wild-type ASFV and the key protein to distinguish wild-type ASFV from CD2v gene-deleted ASFV. It is related to the pathogenicity of the virus. Compared with wild-type ASFV virulent strains, the time of viremia after the weak strain of CD2v knockout infecting the host is prolonged, and clinical symptoms are also reduced. In addition, CD2v can inhibit lymphocyte proliferation and induce immunosuppression (17, 27). In the family of MGF genes, the MGF505 gene is involved in immune escape, can target TANK binding kinase 1 (TBK1), inhibit cGAS-STING-mediated IFN-*β* production, and is an important structural protein for ASFV invasion (28). Partial deletion of the MGF505 gene can reduce viral replication and virulence in alveolar macrophages (1). Therefore, CD2v and MGFs are usually used as targets to design attenuated vaccines and develop diagnostic assays.

Vaccines are always the most effective preventive tools against viral diseases. Since the outbreak of ASF, various approaches have been employed in ASF vaccine design. According to the currently available information, gene deleted live attenuated vaccines (LAVs) generated by rational deleting a single or multiple virulence genes appear to be the most promising vaccine candidates and exhibit a wide range of safety and efficacy against ASF (29–31). Currently, several gene-deleted LAV vaccine candidates have been generated, such as those with the deletion of the CD2v, UK, MGFs and DP148R genes and so on in the ASFV genome (1, 32). However, except for Vietnam, where live attenuated vaccines ASFV-G-∆I177L with deletion of the I177L gene and ASFV-G-∆MGF with deletion of six genes: MGF505-1R, MGF360-12L, MGF360-13L, MGF360-14L, MGF505-2R, and MGF505-3R have been recently commercialized, no registered vaccines are available to prevent and control ASF in other countries (1, 30, 33, 34). Therefore, rapid and specific molecular diagnosis and serological detection by PCR and ELISA, recommended by WOAH, play a pivotal role in preventing and controlling ASF (1). However, naturally gene-deleted lower-virulence ASFV (ASFVΔCD2v/ΔMGF) identified in China and some other country generally cause a chronic and persistent infection course in pigs, lacking typical clinical symptoms compared with wild-type ASFV infections (2, 14, 17–19, 21, 27). The delayed epidemic progression caused by these low-virulent strains results in a postponement or intermittent virus-shedding process in oral and nasal secretions, and the virus is generally undetectable in the blood as well as in nasal and oral secretions after viremia (2, 4, 14). Therefore, ASFV infection cannot be reliably monitored by detecting the virus alone. Regarding antibody detection, the commercial ASFV ELISA kits available can only detect antibody levels in a sample but cannot differentiate serum-positive pigs from wild-type ASFV infection or lower-virulence ASFV with gene deletion. The current published ELISA assays can distinguish between the wild-type and natural CD2v gene-deleted ASFV, but cannot differentiate wild-type and MGF505 gene-deleted ASFV or CD2v and MGF505 double gene-deleted ASFV infection. Therefore, they cannot fully meet the requirements to monitor the current epidemic variation of ASFV. In addition, although there has no commercial vaccine in China, several studies have confirmed that the most potential ASF vaccine candidates are CD2v and MGFs gene-deleted live attenuated vaccines (8, 14, 35, 36). Among these candidates, HLJ/18-7GD with the deletion of seven genes encoding MGF5051R, MGF505-2R, MGF505-3R, MGF360-12L, MGF36013L, MGF360-14L and CD2v, have undergone clinical trials and demonstrated promising potential for vaccine development (29, 37). Therefore, it is imperative to establish an ELISA antibody detection method that can effectively distinguish wild-type ASFV and CD2v and/or MGFs gene-deleted strains.

In this study, the ASFV p30, CD2v, and MGF505 proteins were expressed and purified. A highly sensitive and specific triple protein-based ELISA was developed based on these proteins to detect ASFV antibodies. This newly established ELISA antibody assay can rapidly detect ASFV infection and differentiate the causative virus from wild-type ASFV or CD2v and/or MGF505 gene-deleted strains infection. It offers technical support for the differential diagnosis, epidemiological study of ASFV, and vaccine effectiveness evaluation in the future.

## 2 Materials and methods

### 2.1 Serum samples

Standard ASFV negative (ASFV^−^) and ASFV positive (ASFV^+^) sera were obtained from Qingdao Lijian Bio⁃tech Co. Ltd. (Qingdao, China) and are used to test the optimal concentration of antigen coating and serum dilution; CD2v unexpressed ASFV (ASFV^+^ΔCD2v) positive sera and MGF unexpressed ASFV (ASFV^+^ΔMGF505) positive sera were stored at −80°C in our laboratory and are used to identify the immunogenicity of the proteins ASFV-p30, ASFV-CD2v and ASFV-MGF505. These sera were collected from pigs which were infected by ASFV^+^ΔCD2v or ASFV^+^ΔMGF505 virus strains. The infection status of these pigs was confirmed by detecting ASFV DNA using a commercialized triplex qPCR kit based on p72/CD2v/MGF gene (BioKITai, Xiamen, China) and the serum samples were collected 4 weeks after ASFV infection was verified. Serum samples collected from Pig farms before 2018 (*n* = 30) and from pig farms with no history of ASFV infection from 2019 to 2023 (*n* = 60) are used to determine the threshold value of the assay. These sera were confirmed to be negative for ASFV by a commercialized triplex qPCR kit based on p72/CD2v/MGF gene (BioKITai, Xiamen, China) and a commercialized ELISA kit based on p30 protein (Putai Biology, Luoyang, China). Antibody-positive sera of porcine reproductive and respiratory syndrome virus (PRRSV^+^), porcine pseudorabies virus (PRV^+^), porcine circovirus type 2 (PCV2^+^) and classical swine fever virus (CSFV^+^) were confirmed to be positive by the commercialized ELISA kits (Putai Biology, Luoyang, China) and to be negative for ASFV by a commercialized ELISA kit based on p30 protein (Putai Biology, Luoyang, China) were stored at −80°C in our laboratory. These sera samples were used to validate the analytical specificity of the triple protein-based ELISA. Clinical serum samples (*n* = 59) used to evaluate the precision and test accuracy of ELISA methods with commercially available kits were collected from pig farms suspected of ASFV infection in northern China (including Inner Mongolia Autonomous Region, Shanxi Province, Hebei Province, Shandong Province, etc.) in 2020–2023. These samples were tested by a commercialized triplex qPCR kit based on p72/CD2v/MGF gene (BioKITai, Xiamen, China) and showed 23 ASFV^+^ serum (21 ASFV^+^ wild-type serum, 1 ASFV^+^ΔCD2v serum, 1 ASFV^+^ΔMGF505 serum), and 36 ASFV^−^ serum. These samples were also detected using a commercialized ELISA kit based on p30 protein (Putai biology, Luoyang, China) and showed 24 ASFV^+^ serum, 35 ASFV^−^ serum.

### 2.2 Prokaryotic expression, purification, and immunogenicity identification of recombinant proteins ASFV-p30, ASFV-CD2v, and ASFV-MGF505

The sequences of ASFV p30, CD2v, and MGF505 (ASFV Pig/HLJ/2018, GenBank: MK333180.1) were synthesized by Sangon Biotech (Shanghai, China) and separately cloned into the pET-32a vector through the *EcoR I* and *Xho I* restriction sites (TaKaRa, China). The recombinant plasmids were transformed into competent *Escherichia coli* (*E. coli*) BL21 (DE3) cells after sequence identification and cultured at 37°C. The negative control strains (pET-32a plasmids transformed BL21 cells) were cultured simultaneously. When the culture OD_450nm_ reached 0.7, isopropyl-*β*-D-thiogalactopyranoside (IPTG) (0.5, 1, 1.5, 2%) (Solarbio, Beijing, China) was added to induce protein expression for 2–8 h to optimize protein expression conditions. These proteins were purified by the His-labeled protein purification kit (CWBIO, Jiangsu, China), according to the manufacturer’s instructions.

The endotoxins in purified proteins was removed using a ToxinEraser Endotoxin Removal Kit (Genscript, Nanjing, China) and was detected using a ToxinSensor Chromogenic LAL Endotoxin Assay Kit (Genscript, Nanjing, China) following the manufacturer’s instructions to determine. SDS-PAGE and NanoDrop 2000 were used to analyze the concentration and purity of the purified recombinant proteins ASFV-p30, ASFV-CD2v, and ASFV-MGF505. The yield of expressed proteins was calculated according to the concentration of recombinant proteins. Western blot was used to analyze their antigenicity. Following SDS-PAGE, these proteins were transferred to the PVDF membranes. After overnight sealing with 5% skim milk, the PVDF membranes were incubated with sera against ASFV^+^ wild-type, ASFV^+^ΔCD2v, or ASFV^+^ΔMGF505 (1:2,000) for 3 h. Then, the PVDF membranes were incubated with HRP labeled Rabbit Anti-Pig IgG (H + L) (Biodragon, Beijing, China) (1:5,000) for 3 h. Results were shown by the eECL Western Blot Kit (CWBIO, Jiangsu, China).

### 2.3 Establishment of triple protein-based ELISA method based on recombinant protein ASFV-p30, ASFV-CD2v, and ASFV-MGF505

The purified recombinant proteins ASFV-p30, ASFV-CD2v, and ASFV-MGF505 were used as coating antigens to establish a triple protein-based ELISA to differentiate serum antibodies between wild-type and CD2v /MGF505 gene-deleted ASFV infection. ELISA was carried out on 96-well microtiter plates (BIOFIL, Guangzhou, China). A checkerboard titration of each antigen pool and serum was designed to determine the optimal dilutions of the antigen and serum. The purified recombinant proteins ASFV-p30, ASFV-CD2v, and ASFV-MGF505 were diluted using carbonate buffer (0.159% (w/v) Na_2_CO_3_; 0.293% (w/v) NaHCO_3_) with the range of 4 to 0.125 μg/mL. Microtiter plates were coated with 100 μL per well of the diluted protein and incubated for 1 h at 37°C, then overnight at 4°C. After washed five times with PBST (Solarbio, Beijing, China), the plates were blocked for 3 h at 37°C with 5% skim milk in PBST (Solarbio, Beijing, China) (100 μL/well). Then, the plates were washed as described above, and 100 μL of the serum samples (antibody-positive and-negative standard sera control) that were diluted (from 1:50 to 1:400) with 5% skim milk in PBST was added and incubated for 1 h at 37°C. Subsequently, the 96-well plates were washed and incubated with 100 μL of a 1:5,000 dilution of horseradish peroxidase-conjugated Rabbit Anti-Pig IgG (H + L) per well for 1 h at 37°C. After being washed five times with PBST, the reaction was visualized by incubating the wells with a TMB single component substrate solution (Solarbio, Beijing, China) (100 μL/well) for 10 min at 37°C. The reaction was stopped by adding 50 μL 2 M H_2_SO_4_ per well and the optical density (OD) at 450 nm of each well was measured immediately using an enzyme labeler (Thermo Fisher Scientific, Multiskan FC). All samples were run in triplicate. The optimal antigen and serum concentrations were determined using the criteria that the OD_450 nm_ value of the positive serum was closest to 1.0, together with a maximum positive/negative (P/N) value.

When only the type of blocking solution, the time and temperature of antigen coating, or the dilution concentration of HRP labeled Rabbit Anti-Pig IgG (H + L) were used as the single variable of the ELISA condition, and other steps were the same as above, the optimal blocking solution, antigen coating time and temperature, or the dilution concentration of ELISA were tested. As previously described, the optimal assay conditions were identified as those that yielded the highest P/N value and the ASFV^+^ OD_450 nm_ value closest to 1.0.

### 2.4 Confirmation of the cut-off value and the result criterion

The cut-off value was determined by detecting ASFV^−^ serum samples under the optimal test conditions. The status of these serum samples was confirmed by a commercialized triplex qPCR kit based on p72/CD2v/MGF gene (BioKITai, Xiamen, China) and a commercialized ELISA kit (Putai Biology, Luoyang, China). The OD_450 nm_ value of these ASFV negative serum samples obtained with p30-ELISA, CD2v-ELISA, and MGF505-ELISA was recorded. The cut-off value of each ELISA was established at the mean OD_450 nm_ value (^−^x) + 3 × the standard deviation (SD). The serum sample was considered positive if the OD_450 nm_ value exceeded the cut-off value. Otherwise, it was considered negative. The criteria to determine the triple protein-based ELISA results are presented in Table 1.

**Table 1:** Criteria for the triple protein-based ELISA results.

OD_450 nm_ of p30/CD2v/MGF505 protein-coated well			Determination of results
p30 (>cut-off value)	CD2v (>cut-off value)	MGF505 (>cut-off value)	
Y	Y	Y	ASFV^+^ wild-type
Y	N	Y	ASFV^+^ΔCD2v
Y	Y	N	ASFV^+^ΔMGF505
Y	N	N	ASFV^+^ΔCD2v and ΔMGF505
N	N	N	ASFV^−^

“Y” indicates the OD_450 nm_ of serum samples greater than the cut-off value, and “N” indicates the OD_450 nm_ of serum samples less than the cut-off value.

### 2.5 Evaluation of analytical specificity, analytical sensitivity, repeatability and reproducibility

Serum samples of ASFV^−^, PRRSV^+^, PRV^+^, CSFV^+^, PCV2^+^, standard ASFV^+^, ASFV^+^ΔCD2v, and ASFV^+^ΔMGF505 were detected to determine the analytical Specificity of the developed ELISA at 1:100 dilution. The analytical Sensitivity of the triple protein-based ELISA was evaluated using the standard ASFV^+^ serum diluted from 1: 100 to 1: 3,200. ASFV positive serum samples were tested in the same batch to evaluate the repeatability (intra-assay) of the developed ELISA and were tested at different laboratories to evaluate the reproducibility (inter-assay) of the triple protein-based ELISA.

### 2.6 Comparison with the commercialized kit

A total of 59 clinical serum samples were detected using a commercialized triplex qPCR kit based on p72/CD2v/MGF gene and a commercialized ELISA kit based on p30 protein for a comparison and as a reference method as well. The clinical serum samples were detected using the developed triple protein-based ELISA to calculate the relative diagnostic sensitivity, diagnostic specificity and test accuracy of triple protein-based ELISA method. In this study, we defined relative diagnostic sensitivity (TP/ (TP + FN)) as the ratio of positive tests from the established ELISA to the positive tests by the commercialized triplex qPCR kit, and relative diagnostic specificity (TN/(TN + FP)) was defined as the ratio of negative tests from the established ELISA to the commercialized triplex qPCR kit. Test accuracy = (TP + TN)/(TP + FN + FP + TN) × 100% (Table 2).

**Table 2:** Calculation of test accuracy between triple protein-based ELISA and commercialized qPCR/ELISA kit.

	The triple protein-based ELISA (+)	The triple protein-based ELISA (−)	Total
The commercialized qPCR/ELISA kit (+)	TP	FN	TP + FN
The commercialized qPCR/ELISA kit (−)	FP	TN	FP + TN
Total	TP + FP	FN + TN	TP + FN + FP + TN

TP, the number of the true positive samples; TN, the number of the true negative samples.

FP, the number of the false positive samples; FN, the number of the false negative samples.

### 2.7 Statistical analysis

All experiments were repeated at least three times. SPSS (IBM Corporation, Armonk, NY, USA) and Origin 8.0 (OriginLab, Northampton, MA, USA) were used to perform all statistical tests. All data were presented as the mean + SD. Statistical significance was analyzed using the t-test and was considered at *p* < 0.05.

## 3 Results

### 3.1 Prokaryotic expression, purification, and immunogenicity identification of recombinant proteins ASFV-p30, ASFV-CD2v, and ASFV-MGF505

**Figure 1:**
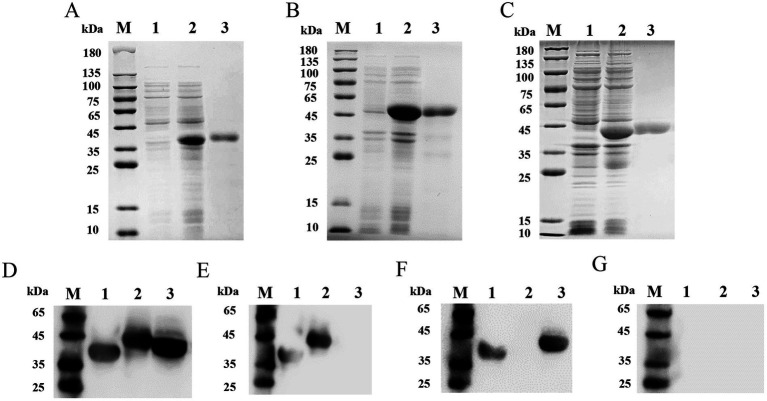
SDS-PAGE and Western blot analysis of purified recombinant proteins ASFV-p30, ASFV-CD2v, and ASFV-MGF505. SDS-PAGE analysis of purified recombinant proteins ASFV-p30, ASFV-CD2v, and ASFV-MGF505 **(A–C)**. The expression and purification of the recombinant proteins ASFV-p30 (39.6 kDa) **(A)**, ASFV-CD2v (46.8 kDa) **(B)**, and ASFV-MGF505 (42.3 kDa) **(C)** were analyzed by SDS-PAGE. Lane M: protein marker; Lane 1: pET-32a; Lane 2: unpurified protein; Lane 3: purified protein. Analysis of immunogenic identification of the ASFV-p30, ASFV-CD2v, and ASFV-MGF505 proteins by Western blot assay **(D–G)**. ASFV-p30, ASFV-CD2v, and ASFV-MGF505 proteins could react with ASFV^+^ serum **(D)**. ASFV-p30 and ASFV-CD2v proteins could react with ASFV^+^ΔMGF505 serum **(E)**. ASFV-p30 and ASFV-MGF505 proteins could react with ASFV^+^ΔCD2v serum **(F)**. ASFV-p30, ASFV-CD2v, and ASFV-MGF505 proteins could not react with ASFV^−^ serum **(G)**. Lane M: protein marker; Lane 1: ASFV-p30; Lane 2: ASFV-CD2v; Lane 3: ASFV-MGF505.

The synthesized protein genes ASFV-p30, ASFV-CD2v, and ASFV-MGF505 were separately cloned into the pET-32a vector and successfully expressed in *E. coli* BL21 cells with the highest expression level under the condition of 1% IPTG induction for 6 h. The calculated molecular weights of the recombinant proteins were 39.6 kDa (Figure 1A), 46.8 kDa (Figure 1B), and 42.3 kDa (Figure 1C), respectively. After purification, highly-purity proteins were obtained. The yield of recombinant Proteins ASFV-p30, ASFV-CD2v, and ASFV-MGF505 was calculated to be 15.28 mg, 11.34 mg, 11.09 mg in 100 mL LB medium. The endotoxins content of these proteins in this study did not exceed 0.1 EU/mg. Western blot analysis was applied to identify purified ASFV-p30, ASFV-CD2v, and ASFV-MGF505. All three recombinant proteins could react with ASFV^+^ sera (Figure 1D) but not with ASFV^−^ sera (Figure 1G). ASFV-p30 and ASFV-CD2v proteins could be recognized by ASFV^+^ΔMGF505 serum, but ASFV-MGF505 protein could not (Figure 1E). ASFV-p30 and ASFV-MGF505 proteins could react with ASFV^+^ΔCD2v serum, but ASFV-CD2v protein could not (Figure 1F). These results indicated that the purified ASFV-p30, ASFV-CD2v, and ASFV-MGF505 proteins had high immunogenicity, and that anti-ASFV-CD2v and anti-MGF505 antibodies present in sera do not cross-react with ASFV-MGF505 and ASFV-CD2v, respectively.

### 3.2 Optimization of experimental conditions for the triple protein-based ELISA

**Figure 2:**
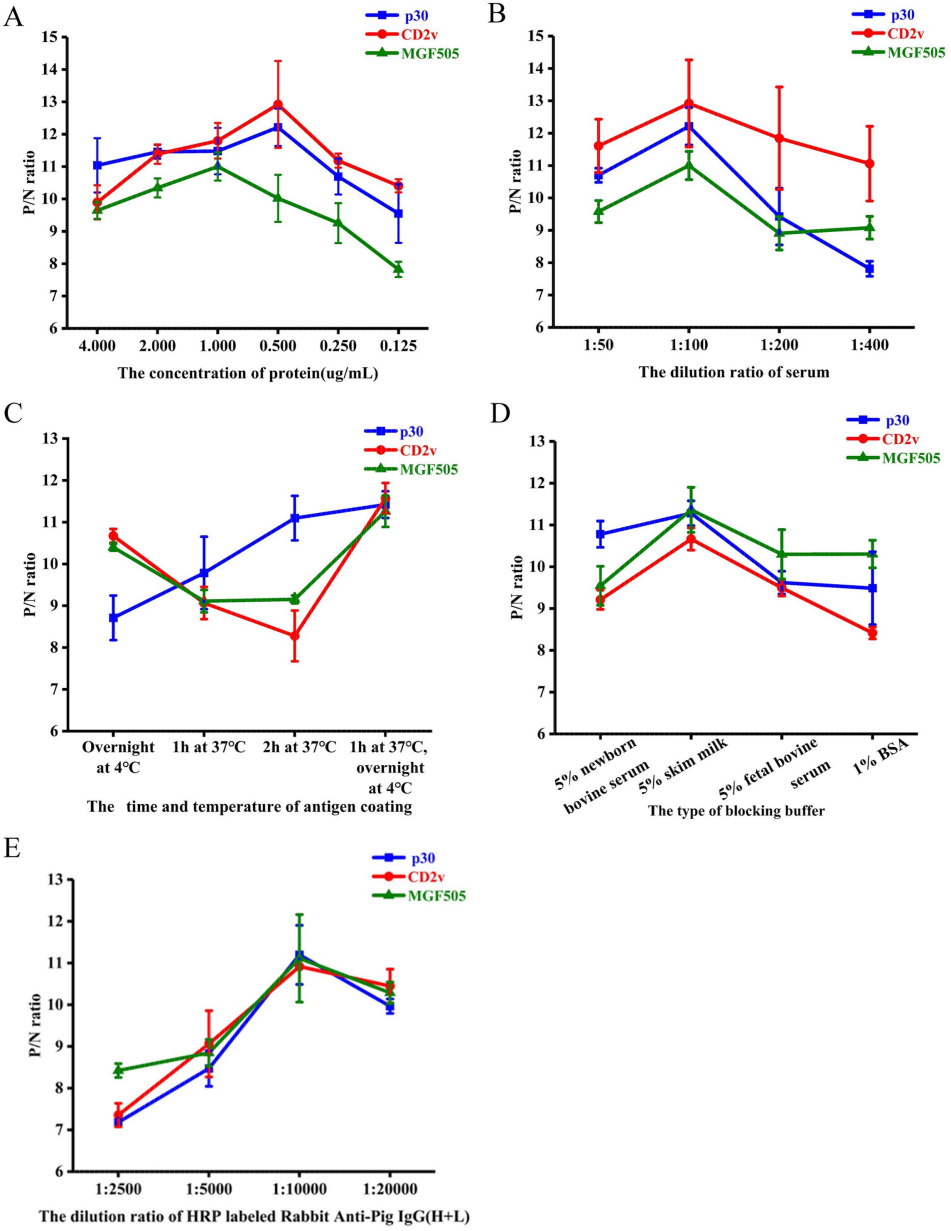
Optimization of experimental conditions for the triple protein-based ELISA. Determination of optimal protein coating concentration. P/N ratios of an ASFV^+^ and ASFV^−^ serum using different concentrations of the ASFV-p30, ASFV-CD2v, and ASFV-MGF505 proteins **(A)**. Determination of optimal serum dilution. P/N ratios of an ASFV^+^ and ASFV^−^ serum with various dilutions of serum samples **(B)**. Determination of optimal antigen coating conditions. P/N ratios of an ASFV^+^ and ASFV^−^ serum with various coating times and temperatures of each antigen **(C)**. Determination of the best blocking buffer. P/N ratios of an ASFV^+^ and ASFV^−^ serum with various blocking buffer **(D)**. Determination of the optimal dilution of the HRP labeled Rabbit Anti-Pig IgG (H + L). P/N ratios of an ASFV^+^ and ASFV^−^ serum with various dilutions of HRP labeled Rabbit Anti-Pig IgG (H + L) **(E)**. P/N ratio data represent mean ± SD.

The optimal antigen concentration and serum sample dilution were determined using checkerboard titration. The maximum value of P/N was obtained when the concentration of the ASFV-p30 and ASFV-CD2v protein was 0.5 μg/mL, ASFV-MGF505 protein was 1 μg/mL (Figure 2A) and the serum dilution was 1:100 (Figure 2B). The optimal antigen coating time and temperature was 37°C for 1 h and then overnight at 4°C (Figure 2C). The best blocking buffer was PBST with 5% skim milk (Figure 2D), and the optimal dilution of HRP labeled Rabbit Anti-Pig IgG (H + L) was 1:10,000 (Figure 2E).

### 3.3 Confirmation of the cut-off value

**Figure 3:**
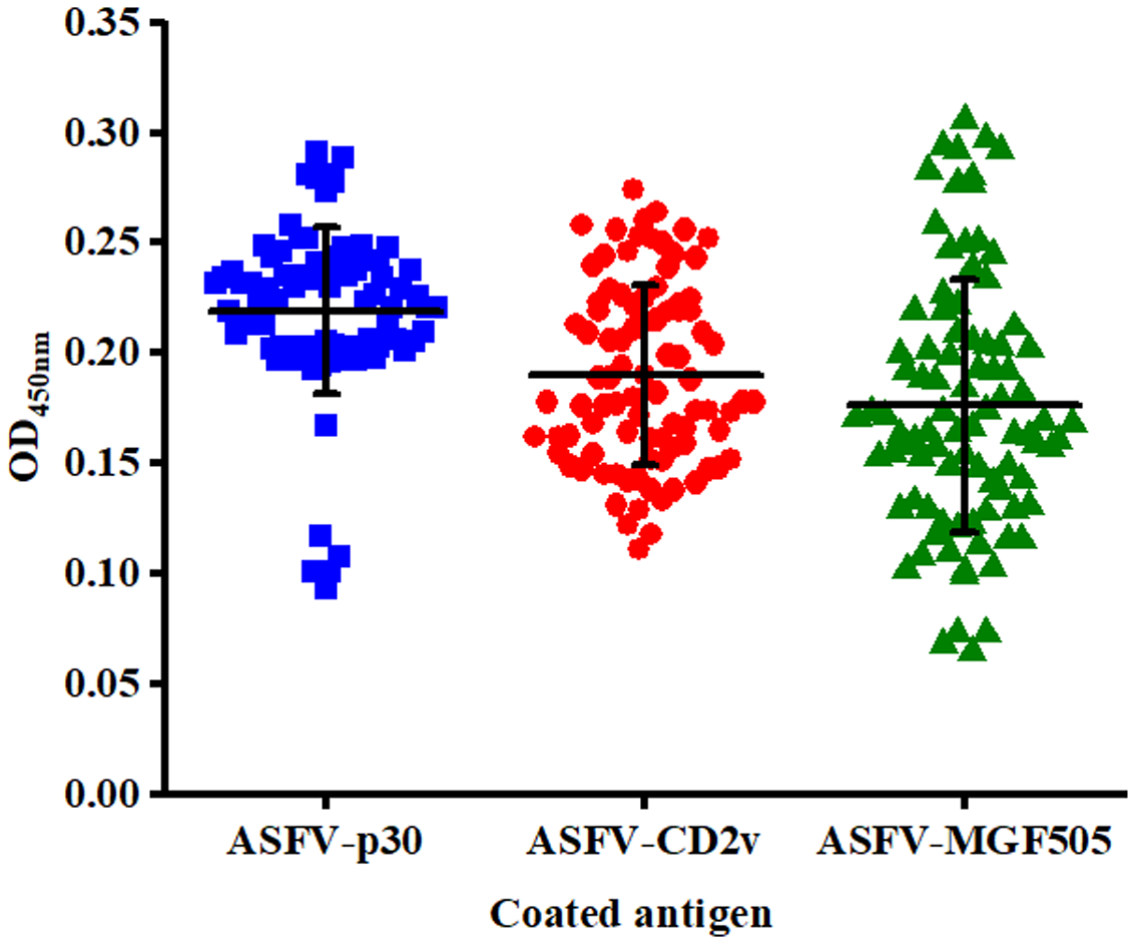
Confirmation of the cut-off value for the triple protein-based ELISA. ASFV^−^ serum samples (*n* = 90) were tested by the triple protein-based ELISA to determine the cut-off value under the optimal test conditions. The cut-off value of each ELISA was established at the mean OD_450 nm_ value (^−^x) + 3 × the standard deviation (SD).

The cut-off value was determined by detecting serum samples negative for ASFV under optimal conditions. The mean OD_450 nm_ value of ELISA based on the protein ASFV-p30, ASFV-CD2v, and ASFV-MGF505 was 0.219, 0.190, and 0.176, with an SD of 0.038, 0.041, and 0.057, respectively. Therefore, the cut-off value of the ELISA based on the protein ASFV-p30, ASFV-CD2v, and ASFV-MGF505 was calculated to be 0.333, 0.313, and 0.347 (mean OD_450 nm_ value of negative samples plus three SD), respectively (Figure 3).

### 3.4 Evaluation of analytical specificity, analytical sensitivity, repeatability and reproducibility

**Figure 4:**
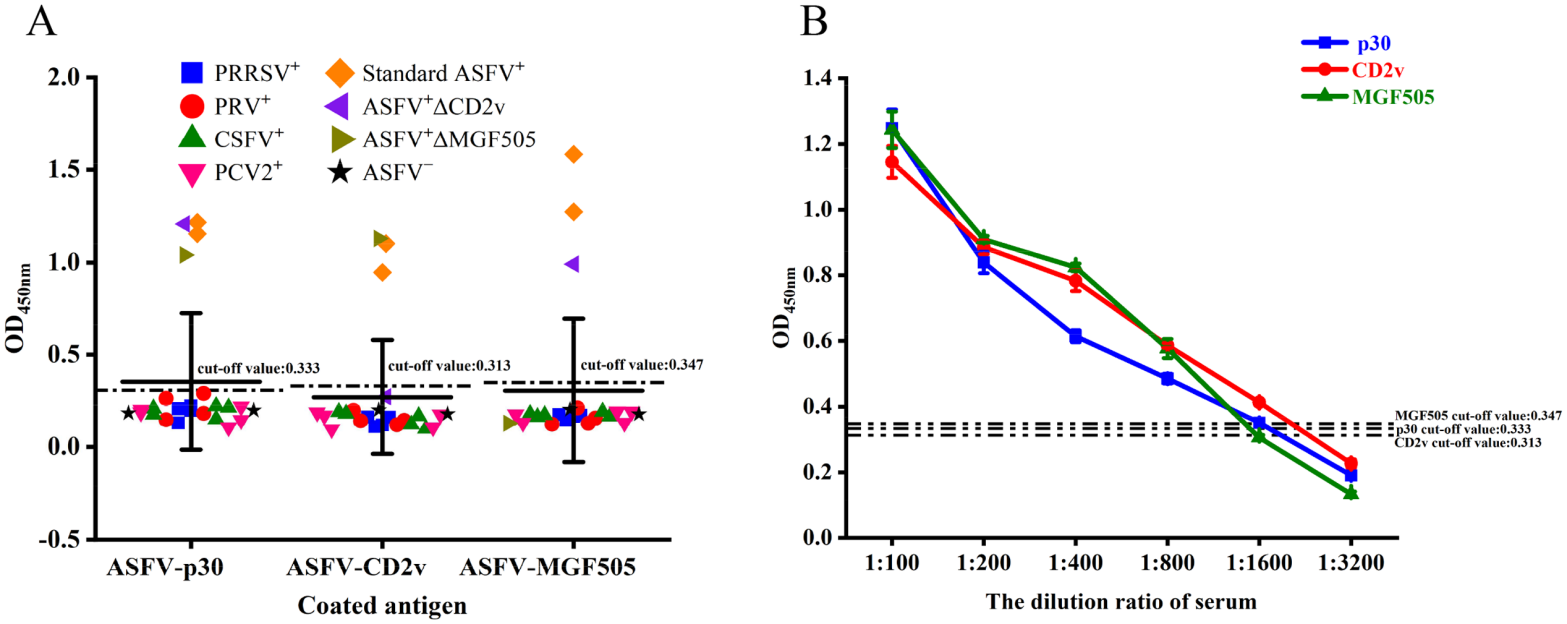
Determination of the analytical specificity and analytical sensitivity of the triple protein-based ELISA. The triple protein-based ELISA cannot detect ASFV^−^, PRRSV^+^, PRV^+^, CSFV^+^, and PCV2^+^ serum samples, but can detect standard ASFV^+^, ASFV^+^ΔCD2v, and ASFV^+^ΔMGF505 serum samples at 1:100 dilution **(A)**. Standard serum ASFV^+^ was diluted to test the detection limit of triple protein-based ELISA. According to the cut-off value, the ELISA based on the ASFV-p30 and ASFV-CD2v proteins could detect a 1,600-fold dilution of standard serum ASFV^+^, and the ELISA based on the ASFV-MGF505 protein could detect a 800-fold dilution of standard serum ASFV^+^. The dashed lines indicate the cut-off value **(B)**.

The analytical specificity of the triple protein-based ELISA method was evaluated by detecting the reactivity of each purified protein with antibodies against ASFV^−^, PRRSV^+^, PRV^+^, CSFV^+^, PCV2^+^, standard ASFV^+^, ASFV^+^ΔCD2v, and ASFV^+^ΔMGF505. Only the standard ASFV^+^ (the mean OD_450 nm_ ± SD: 1.216 ± 0.007, 1.154 ± 0.044), ASFV^+^ΔCD2v (the mean OD_450 nm_ ± SD: 1.208 ± 0.014), and ASFV^+^ΔMGF505 (the mean OD_450 nm_ ± SD: 1.041 ± 0.054) serum samples were positive in ASFV-p30 protein-coated ELISA; standard ASFV^+^ (the mean OD_450 nm_ ± SD: 1.101 ± 0.014, 0.947 ± 0.043) and ASFV^+^ΔMGF505 (the mean OD_450 nm_ ± SD: 1.129 ± 0.012) were positive in ASFV-CD2v protein-coated ELISA; and standard ASFV^+^(the mean OD_450 nm_ ± SD: 1.273 ± 0.054, 1.583 ± 0.069) and ASFV^+^ΔCD2v (the mean OD_450 nm_ ± SD: 0.991 ± 0.059) serum samples were positive in ASFV-MGF505 protein-coated wells. On the contrary, ASFV^−^ serum samples and other pathogen positive sera were negative in the triple ELISA. Overall, the triple protein-based ELISA assays were an effective method for detecting and distinguishing ASFV antibodies (Figure 4A).

The analytical sensitivity of the triple protein-based ELISA method was assessed by detecting serial dilutions of standard ASFV^+^ serum samples. Both the ASFV-p30 and ASFV-CD2v protein-based ELISA could detect a 1,600-fold dilution of standard ASFV^+^ serum, while the ELISA method based on the ASFV-MGF505 protein could detect an 800-fold dilution of standard ASFV^+^ serum, revealing that the detection limit of the triple ELISA was 1: 800 (Figure 4B).

ASFV positive serum samples were analyzed to validate the repeatability and reproducibility of the triple protein-based ELISA method. The CV values of intra-assay and inter-assay were below 5% (Table 3), indicating the good repeatability and reproducibility of the triple protein-based ELISA.

**Table 3:** Repeatability and reproducibility test of the triple protein-based ELISA.

Assay	Serum samples	ASFV-p30		ASFV-CD2v		ASFV-MGF505	
		^−^x ± SD	CV%	^−^x± SD	CV%	^−^x ± SD	CV%
Intra-assay	S1	1.150 ± 0.039	3.391	1.124 ± 0.026	2.313	1.347 ± 0.041	3.044
	S2	1.247 ± 0.048	3.849	1.220 ± 0.050	4.098	1.244 ± 0.042	3.376
	S3	1.069 ± 0.043	4.022	0.887 ± 0.022	2.480	1.000 ± 0.038	3.800
	S4	1.159 ± 0.056	4.832	1.230 ± 0.040	3.252	0.195 ± 0.009	4.615
	S5	1.130 ± 0.040	3.540	0.915 ± 0.011	1.202	0.933 ± 0.015	1.608
Inter-assay	S1	1.123 ± 0.012	1.069	1.165 ± 0.041	3.519	1.311 ± 0.024	1.831
	S2	1.250 ± 0.059	4.720	1.181 ± 0.055	4.657	1.258 ± 0.022	1.749
	S3	1.109 ± 0.034	3.066	0.927 ± 0.041	4.423	1.089 ± 0.048	4.408
	S4	1.173 ± 0.029	2.472	1.219 ± 0.055	4.512	0.194 ± 0.009	4.639
	S5	1.127 ± 0.043	3.815	0.942 ± 0.034	3.609	0.948 ± 0.022	2.321

### 3.5 Comparison with the commercialized kit

**Figure 5:**
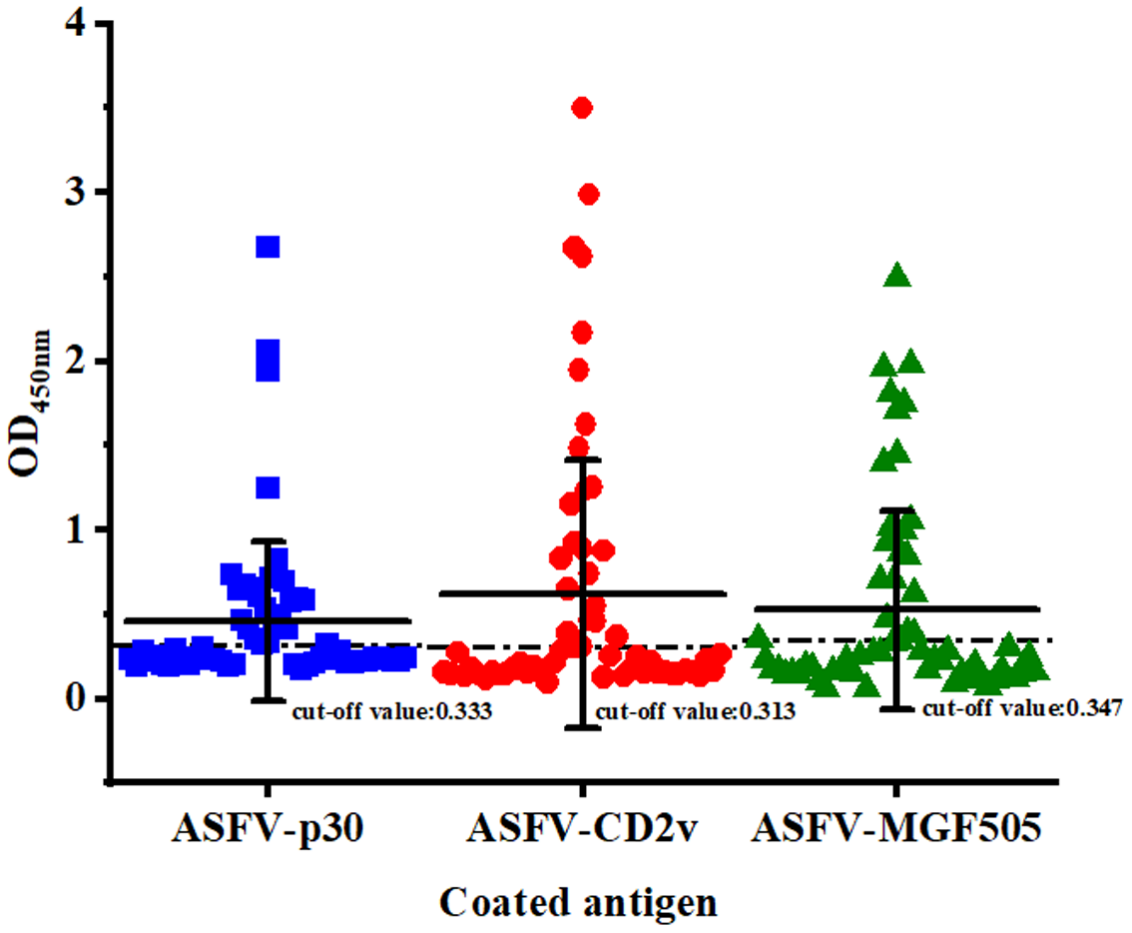
Clinical serum samples (*n* = 59) detection results of the triple protein-based ELISA.

Clinical serum samples (*n* = 59) were tested by a commercialized triplex qPCR kit, commercialized ELISA kit and the triple protein-based ELISA to calculate the diagnostic sensitivity, diagnostic specificity and test accuracy of established triple protein-based ELISA method. The triple protein-based ELISA identified a total of 22 ASFV^+^ sera (19 ASFV^+^ wild-type sera, 1 ASFV^+^ΔCD2v serum, 2 ASFV^+^ΔMGF505 sera), and 37 ASFV^−^ sera (Figure 5). Compared with the commercialized triplex qPCR kit, the relative diagnostic sensitivity of the triple protein-based ELISA based on the protein ASFV-p30 was 95.65% (95% confidence interval: 78.05 to 99.89%) among the ASFV^+^ serum, and the relative diagnostic specificity of the method was 97.30% (95% confidence interval: 85.84 to 99.93%) among the ASFV^−^ serum; the relative diagnostic sensitivity of the triple protein-based ELISA based on the protein ASFV-CD2v was 95.45% (95% confidence interval: 77.16 to 99.89%) among the ASFV^+^ serum, and the relative diagnostic specificity of the method was 97.37% (95% confidence interval: 86.19–99.93%) among the ASFV^−^ serum; the relative diagnostic sensitivity of the triple protein-based ELISA based on the protein ASFV-MGF505 was 90.91% (95% confidence interval: 70.84–98.88%) among the ASFV^+^ serum, and the relative diagnostic specificity of the method was 94.87% (95% confidence interval: 82.68–99.37%) among the ASFV^−^ serum. The test accuracy of the triple protein-based ELISA and commercialized triplex qPCR kit was 98.31% (58/59). Compared with the commercialized ELISA kit, the test accuracy was 96.61% (57/59). These results indicating that the method can diagnose ASF and differentiate serum antibodies from wild-type ASFV from CD2v/MGF505 unexpressed ASFV infection (Table 4). Compared with the commercialized ELISA kit available and published ELISA methods, the triple protein-based method overcomed the current limitation of not being able to distinguish wild-type and MGF505 gene-deleted ASFV or CD2v and MGF505 double gene-deleted ASFV infection. It is of great significance to distinguish ASFV infected strains in pig farms and take corresponding prevention and control measures.

**Table 4:** Comparison of the triple protein-based ELISA with the commercialized triplex qPCR kit and the commercialized ELISA kit.

	The triple protein-based ELISA (+)	The triple protein-based ELISA (−)	Total
The commercialized triplex qPCR kit (+)	22	1	23
The commercialized triplex qPCR kit (−)	0	36	36
Total	22	37	59
The commercialized ELISA kit (+)	22	2	24
The commercialized ELISA kit (−)	0	35	35
Total	22	37	59

## 4 Discussion

ASFV is progressively spreading throughout the world, which has substantial economic implications for the global pig industry (14). The emergence of gene-deleted ASFV strains characterized by longer incubation and high transmission ability increased the difficulty of ASF control (27). Because there are no effective anti-ASFV drugs and commercialized ASFV vaccines available, cutting off transmission routes by strict biosecurity measures, together with effective detection for early diagnosis and elimination of infected pigs, are currently the primary strategy for prevention and control (3, 4, 38).

Many methods have been developed to diagnose ASFV infection by detecting ASFV or ASFV-specific antibodies. Among these detection methods, fluorescence quantitative PCR (qPCR) and ELISA are considered the main methods owing to the advantages of high throughput, specificity, and sensitivity (38). For the detection of ASFV, several single or multiplex qPCRs based on different genes have been reported, except for commercialized qPCR kits (39, 40). All these reported detection methods can effectively detect ASFV in positive samples. In addition, some assays can also differentially detect wild-type and gene-deleted ASFV (39, 40). However, the postponed or discontinuous virus shedding process caused by the gene-deleted low-virulent strains which often result in false negative detection results in some ASFV infected cases (4). In addition, ASF was classified as a first-class animal infectious disease in China (4). These molecular diagnostic methods, which use ASFV nucleic acid as the detected target, have stringent requirements for biosafety facilities, diagnostic personnel, and the viral genome extraction process (4, 38, 41). Compared with qPCR, ELISA does not have strict requirements because there is no need to handle viruses. Furthermore, in the absence of a vaccine, specific antibodies in the serum indicate virus infection, and these antibodies can persist for a long time in infected pigs (4, 15, 41, 42). Therefore, antibody detection by serological assays is essential for the prevention and control of ASF, especially for subacute and chronic ASF (15).

Currently, using p30, p32, p54, p62, p72, and other viral proteins, several single, double, triple, or quadruple antigen-based ELISA assays were established for the detection of ASFV antibodies, and some ELISA kits have been commercialized (15, 16, 25, 38, 43–45). These methods can accurately distinguish the serum-positive pig from the negative one but cannot differentiate the serum-positive pigs infected with wild-type or gene-deleted ASFV. To identify wild-type and CD2v-deleted strains of ASFV, an indirect ELISA based on the extracellular domain of the expressed CD2v protein has been established (17). This method does not have cross-reaction with serum samples infected with CD2v deleted ASFV, PRRSV, CSFV, PCV, PRV, swine FMDV, and PEDV. The serum dilution that can be identified was 1:2,560. The coefficient of variation in and between batches was <10%, and the compliance rate was 99.4% (17). However, this indirect ELISA only uses the extracellular domain of the CD2v protein as the detection protein. When the detected result is negative, it is hard to determine whether the pig was not infected with ASFV or infected with CD2v gene-deleted ASFV using this ELISA method alone. To meet this requirement, this assay should be used in combination with traditional ELISA simultaneously. In order to differentiate wild-type and CD2v gene-deleted ASFV, a dual ELISA based p30 and CD2v protein was established (14). The dual ELISA showed excellent specificity without cross-reactions with antibodies of PRRSV, CSFV, JEV, PRV, or PPV, and high sensitivity with a maximum detect dilution of the ASFV-infected positive serum samples of 5,120 times (14). Another dual indirect ELISA based on p54 and CD2v proteins has been developed to specifically distinguish serum antibodies from pigs infected with wild-type ASFV or possessing attenuated vaccine candidate HLJ/18-7GD immunization (42). The results showed that the diagnostic method has excellent specificity and good reproducibility. It can not only effectively distinguish antibodies induced by wild-type virulent ASFV infection from the vaccine candidate HLJ/18-7GD immunization, but also appropriate to differentiate wild-type and natural CD2v gene-deleted ASFV infection (42). Recently, two ASFV-p72 and -CD2v nanobody-based competitive ELISAs (cELISAs) were developed to detect anti-ASFV antibodies. The two assays showed high sensitivity, specificity, reproducibility, and stability and their combination could differentiate pigs infected with wild-type and CD2v-deleted ASFV (36). However, although these dual ELISA methods have high specificity and sensitivity and are appropriate for differentiating a wild-type and CD2v deleted ASFV infection, they cannot differentiate wild-type and MGF505 gene-deleted ASFV or CD2v and MGF505 double-gene-deleted ASFV infection. Furthermore, they cannot distinguish MGF505 gene-deleted ASFV from CD2v and MGF505 double-gene-deleted ASFV.

The ASFV p30 protein is a crucial structural component that exhibits early expression and phosphorylation in infected cells, showing robust immunogenicity. The p30 protein was reported to be one of the most antigenic ASFV proteins and is generally used as an antigen to develop a serological diagnosis (38, 46). The ELISA detection method using p30 as the coating antigen can basically be used for the whole process of monitoring after ASFV infection (25) and some have been commercialized, such as the commercial ELISA kits (Svanovir, Uppsala, Sweden) and (Putai biology, Luoyang, China).

This study expressed the proteins ASFV-p30, ASFV-CD2v, and ASFV-MGF505 in *E. coli* and purified these proteins by the His-labeled protein purification kit. After purity testing and endotoxin removal, using these proteins as coating antigens, a triple protein-based ELISA assay was developed. The triple protein-based ELISA realized the differentiation detection of different ASFV infection strains by coating p30 protein expressed by all ASFV strains, CD2V protein not expressed in ASFV^+^ΔCD2v strains, and MGF505 protein not expressed in ASFV^+^ΔMGF505 strains. The assay overcomed the limitations of the commercialized ELISA kit available and published ELISA methods that cannot distinguish MGF505 gene-deleted ASFV from the wild-type, and the CD2v and MGF505 double-gene-deleted ASFV. The steps of recombinant protein purification and endotoxin removal procedure mitigated the potential contamination with bacterial proteins and endotoxins due to the use of a prokaryotic expression system (*E. coli*), thus reduced the false-positive results of the developed ELISA. It was known that the dilution of testing sera, the reaction regents and reaction time of each process are usually different between different ELISA kits, each need a separately operation which was complex and time consuming. In this study, although each protein of this triple ELISA is independent detection of ASFV virus antibodies against the corresponding proteins, the optimal reaction conditions of the three detection methods are consistent with each other after optimized. Therefore, ASFV-P30, ASFV-CD2V and ASFV-MGF505 proteins can be separately coated on the same microtiter plates and detect the corresponding antibodies simultaneously under the same reaction conditions, which simplified the differential detection process and saved detection time. This newly established triple protein-based ELISA showed good specificity, sensitivity, repeatability and reproducibility. The repeatability and reproducibility exceed those of ASFV ELISA methods established both by Wang et al. (CV value was <20%) and Jiang et al. (CV value was <10%) (17, 42). To evaluate test accuracy and validate whether the newly developed methods could be used to differentiate wild ASFV infection and gene-deleted ASFV infection, a total of 59 clinical serum samples were detected by this triple protein-based ELISA, commercialized triplex qPCR kit based on p72/CD2v/MGF gene (BioKITai, Xiamen, China) and commercialized ASFV antibody detection kit (Luoyang Putai Biological Technology Co., Ltd., China) which use p30 protein as detecting antigen. The results of the triple protein-based ELISA showed that 22 (19 were ASFV^+^ wild-type, one was ASFV^+^ΔCD2v, and two were ASFV^+^ΔMGF505) of the 59 samples were positive for the ASFV antibody. This indicates that wild-type and gene-deleted ASFV strains were simultaneously circulating in northern China. Compared with the detection results of the commercialized triplex qPCR kit (21 ASFV^+^ wild-type sera, 1 ASFV^+^ΔCD2v serum, 1 ASFV^+^ΔMGF505 serum, and 36 ASFV^−^ sera), the test accuracy was 98.31% (58/59). Compared with the detection results of the commercialized ELISA kit (24 ASFV^+^ sera and 35 ASFV^−^ sera), the test accuracy was 96.61% (57/59). These results indicate that the triple protein-based ELISA method not only can effectively discriminate the antibodies induced by wild-type ASFV infection from ASFV∆CD2v virus strain infection but can effectively differentiate among wild-type ASFV, ASFV∆CD2v virus strain, and/or ASFVΔMGFs virus strains infection. When further analysis the detection results of these assays, we found that one of the samples was detected to be ASFV^+^ΔMGF505 by the triple protein-based ELISA, ASFV^+^ by the commercial ELISA kit, but ASFV^−^ by the commercialized triplex qPCR kit. When the PCR detection of the serum sample yields a negative result, the pig either not be infected with ASFV or has infected with lower virulent ASFV and was in the stage of convalescence/virus non-shedding of the disease. The latter is quite likely, since the positive results of the commercial ELISA and triple ELISA have proved it. Therefore, the triple protein-based ELISA can detect the PCR false negatives due to the postponed or discontinuous virus shedding in naturally gene-deleted strains and can differentiate ASFV^+^ΔMGF505 from the wild-type. Compared with other commercialized ELISA kits and published ELISA methods, this method is more suitable for the current prevalence of ASFV. It offers an important diagnostic tool for the effective prevention and control of ASFV.

In conclusion, in this study a triple protein-based ELISA method was established based on the proteins ASFV-p30, ASFV-CD2v, and ASFV-MGF505. It was sensitive and specific, and can effectively differentiate the antibodies induced by wild-type ASFV from the gene-deleted ASFV. It will be useful for the serological differential diagnosis and epidemiology study of ASFV. At the same time, the p30, CD2v and MGF505 proteins were considered the key research targets for attenuated and subunit vaccines. Therefore, in the future, this newly developed triple ELISA method can also be used to discriminate vaccine immunization from natural infection. However, because the clinical samples used to evaluate the applicability of the developed ELISA were all collected from suspected ASFV-infected pig farms, therefore, geographical regions and the numbers of samples are limited, more samples from different geographical regions at home and abroad should be collected to further evaluate the newly developed triple protein-based ELISA method to ensure global applicability and consistency before commercialization.

## Data Availability

The original contributions presented in the study are included in the article/supplementary material, further inquiries can be directed to the corresponding authors.
